# Influence of a Training Academy on the Parasympathetic Nervous System Reactivation of Firefighter Recruits—An Observational Cohort Study

**DOI:** 10.3390/ijerph18010109

**Published:** 2020-12-26

**Authors:** David J. Cornell, Sabrina E. Noel, Xiyuan Zhang, Kyle T. Ebersole

**Affiliations:** 1 Health Assessment Laboratory, University of Massachusetts Lowell, Lowell, MA 01854, USA; sabrina_noel@uml.edu; 2 Center for Population Health, University of Massachusetts Lowell, Lowell, MA 01854, USA; xiyuan_zhang@uml.edu; 3 Department of Physical Therapy and Kinesiology, University of Massachusetts Lowell, Lowell, MA 01854, USA; 4 Department of Biomedical and Nutritional Sciences, University of Massachusetts Lowell, Lowell, MA 01854, USA; 5 Human Performance and Sport Physiology Laboratory, University of Wisconsin-Milwaukee, Milwaukee, WI 53211, USA; ebersole@uwm.edu; 6 Department of Occupational Sciences and Technology, University of Wisconsin-Milwaukee, Milwaukee, WI 53211, USA

**Keywords:** heart rate recovery, autonomic nervous system function, tactical athletes

## Abstract

Sudden cardiac death (SCD) is the leading type of line-of-duty death among firefighters. An inability to restore parasympathetic nervous system (PSNS) control after activity is associated with SCD. Post-exercise heart rate recovery (HRR) provides unique insight into reactivation of the PSNS. Thus, the purpose of this study was to examine longitudinal changes in HRR responses of 25 male firefighter recruits. HR data were collected after submaximal exercise at week 1 (W1), week 6 (W6), and week 15 (W15) of their training at an academy. Percent maximal heart rate (%MHR) measures were computed at each HRR time point (%MHR_0_, %MHR_15_, %MHR_30_, %MHR_45_, %MHR_60_, %MHR_120_, %MHR_180_) and absolute HRR values were calculated at 30 s (ΔHRR_30_), 60 s (ΔHRR_60_), 120 s (ΔHRR_120_), and 180 s (ΔHRR_180_). After controlling for age and percent body fat, there was no statistically significant interaction between Week × HRR (*p* = 0.730), and there were no changes in ΔHRR_30_, ΔHRR_60_, and ΔHRR_120_, and ΔHRR_180_ indices across time. However, %MHR at W6 and W15 was significantly lower than %MHR at W1 at every HRR time point (*p*s < 0.001). Therefore, although the firefighter recruit training academy elicited positive training adaptations, changes in PSNS reactivation after submaximal activity were not identified.

## 1. Introduction

The occupation of firefighting requires intense physical exertion [1], placing extreme cardiovascular strain on a firefighter [2], with heart rate responses that are near [3], and sometimes exceeding [4], an individual’s predicted maximal heart rate. Due to this intense cardiovascular strain, it is not surprising that sudden cardiac deaths (SCDs) have accounted for the greatest proportion of firefighter fatalities in the United States (U.S.) in almost every year since statistics started being recorded by the National Fire Protection Association (NFPA) in 1977 [5]. In addition, for every SCD, there are an estimated 17-25 additional non-fatal line-of-duty cardiac events (stroke, heart attack, etc.) [1], with 19 additional cardiac events occurring in 2017 alone [5,6]. While the majority of these SCDs occur during, or shortly after, responding to fire suppression activities [7], previous research has also demonstrated that the odds of a firefighter experiencing a SCD after returning from a call remain 2.2 to 10.5 times higher than during nonemergency duties [8]. These results indicate that even after completing the physiologically demanding tasks of firefighting, the risk of SCD among firefighters remain elevated for a period of time after the call, suggesting that firefighters may not be able to achieve proper physiological recovery after these strenuous fire suppression calls, placing them at risk for cardiac incidents for an extended period of time.

Based on the excessively high heart rate response achieved during fire suppression activity, it has been hypothesized that inappropriately elevated sympathetic nervous system (SNS) activation after fire suppression activities may be contributing to this elevated risk of SCD [2]. Post-exercise heart rate recovery (HRR) is a commonly utilized tool to examine sympathetic and parasympathetic branches of the autonomic nervous system (ANS) [9]. Since the initial reduction in heart rate after exercise is largely due to reactivation of the parasympathetic nervous system (PSNS) via input from the vagus nerve [10,11], HRR is considered to be a non-invasive and clinically useful metric to examine the ability of the PSNS to regain control of the sinoatrial node of the heart after physical exertion [9]. In addition, previous research has demonstrated that diminished HRR (≤12 b·min^−1^) during the first minute after exercise is an independent predictor of all-cause and cardiovascular mortality [12,13], including among asymptomatic individuals [14,15]. It has subsequently been suggested that PSNS control may produce an “antiarrhythmic effect” by prolonging ventricular refractoriness [16]. Since it has been hypothesized that arrhythmias have contributed to on-duty firefighter SCDs [17], it is possible that increasing the HRR of firefighters may result in greater cardio-protection for these at-risk individuals [10].

Previous research has examined the longitudinal changes in health and fitness that occur within firefighter academy training programs. In general, these academies have demonstrated an ability to create positive adaptations in body composition, aerobic fitness, muscular power, strength, and endurance [18,19,20], theoretically placing these individuals at a lower risk of injury as they enter active-duty service [21]. However, there has been a lack of research examining potential changes in post-exercise ANS recovery as firefighter recruits progress through these academies. Given the link between HRR and all-cause mortality [12,13,14,15], and the previously identified SCD issues within the firefighter population [5,6], investigation of the ability of these academies to elicit positive adaptations in HRR among firefighter recruits is warranted. Accordingly, the purpose of the current study was to examine longitudinal changes in HRR responses of firefighter recruits during the course of their firefighter academy training program.

## 2. Materials and Methods

### 2.1. Participants

Participants were recruited from the same Midwest U.S. urban fire department. Participants were recruited via in-person informational sessions presented by researcher study staff and were provided opportunities to ask questions regarding study purpose and procedures. Participants were considered eligible to participate if they were older than 18 years of age and were cleared for full participation within their training academy. Participants were eligible if they (1) were not taking any prescribed medication for a symptomatic illness; (2) did not sustain an injury or have surgery on their knees, hips, or ankles in the last year; (3) had not been previously diagnosed with a heart condition or had not experienced chest pain or dizziness during exercise; (4) were not currently pregnant; and/or (5) had not been instructed by a physician to refrain from participating in exercise or physical activity.

Based on these criteria, all recruited participants were eligible to participate and a convenience sample of 25 male firefighter recruits volunteered to participate in the current study. Participant characteristics at baseline are presented in Table 1. All participants were cleared by their fire department for full participation within their firefighter training academy. This study was conducted in accordance with the Declaration of Helsinki and was approved by the Institutional Review Board at the University of Wisconsin-Milwaukee (Protocol Number: 13.180). All participants provided written informed consent before data were collected.

### 2.2. Study Design

This study characterized longitudinal changes in ANS recovery after submaximal exercise among a cohort population of firefighter recruits as they progressed through the same 16-week firefighter academy training program. This was accomplished by observationally examining changes in HRR profiles at week 1 (W1), week 6 (W6), and week 15 (W15). Due to the scheduling needs of the fire department, it was not possible to collect data during the middle or final week of the academy training program, and thus, data were collected during W6 and W15 to represent the mid-point and end of the academy program, respectively.

#### Firefighter Academy Training Program

All participants completed the same firefighter academy training program with an urban fire department that had previously adopted *The Fire Service Joint Labor Management Wellness-Fitness Initiative (WFI)*, which was jointly developed by the International Association of Fire Fighters (IAFF) and the International Association of Fire Chiefs (IAFC) [22]. Based on this programming, firefighter recruits completed training programming 8 h per day, 5 days per week, during this training academy.

Structured physical training regarding mastery of the technical skills associated with firefighting (e.g., ladder raising, victim rescue, roof ventilation, etc.) was completed on a daily basis, with total time varying from 1–2 h per day. Structured exercise programming, that included both aerobic exercise and total body resistance training sessions also occurred each morning. In addition, general health education classes related to physical fitness, nutrition, and stress management, were concurrently integrated into this physical training programming. Researchers of the current study did not control or influence the implementation of this training program, and the program was consistent with methods previously employed by the training academy of the respective fire department, as well as with other training programs implemented in the firefighter literature [18,19,23].

### 2.3. Procedures

All data were collected indoors in a group testing format in the gymnasium of the training academy facility associated with the fire department of the firefighter recruits. All participants wore athletic clothing (i.e., t-shirt, athletic shorts, and athletic shoes) and data were collected in the morning (between the hours of 1000 and 1100). All data were collected in the same manner at each week (W1, W6, W15) and there were no participants who dropped out of the study.

#### 2.3.1. Body Composition Data

Percent body fat (BF) of each participant was estimated using skinfold assessment techniques commonly utilized for the firefighter population in the scientific literature [18,24]. Specifically, skinfolds measures (mm) were taken from the right pectoral, triceps, and subscapular locations using a Lange skinfold caliper (Beta Technology, Santa Cruz, CA, USA) [25]. Body density was then calculated using the Jackson and Pollock three-site equation for males [26], and based on these body densities, BF (%) was determined using the corresponding Siri equation [27]. All skinfold data were collected by the same researcher across all participants (K.T.E.).

#### 2.3.2. Heart Rate Data

As part of normal fitness testing within the training academy, all participants completed the Forestry Step Test [28], which has been previously utilized among the firefighter population [18,19,29]. This testing protocol requires participants to step up and down on a 40 cm box to the beat of a metronome set to 90 beats per minute (b·min^−1^) for five minutes. Upon finishing this test, participants sat quietly on the box for a total of a three minutes. During this recovery period, heart rate (b·min^−1^) data was recorded upon immediately finishing this test (HR_0_), as well as 15 s (HR_15_), 30 s (HR_30_), 45 s (HR_45_), 60 s (HR_60_), 120 s (HR_120_), and 180 s (HR_180_) post-test. All heart rate data were collected using Polar T31i heart rate monitors (Polar Electro, Lake Success, NY, USA). There were no missing or invalid data. Previous research has demonstrated excellent validity (*r* = 0.976–1.00) [30] and test-retest reliability (ICCs = 0.93–0.95) [31] of these monitors when collecting heart rate data during exercise and good reliability of raw heart rate values collected after submaximal exercise (ICCs = 0.68–0.80) [32].

The overall HRR responses were characterized by calculating percent maximal heart rate (%MHR) of each participant’s raw heart rate value at each HRR time point (%MHR_0_, %MHR_15_, %MHR_30_, %MHR_45_, %MHR_60_, %MHR_120_, %MHR_180_) during each week (W1, W6, W15). Specifically, heart rate data were normalized to each participant’s age-predicted maximal heart rate based on a previously validated equation provided by Tanaka et al. [33]:

%MHR = 208 − (0.7 × age [yrs])(1)

In addition, the PSNS reactivation of each participant was characterized by calculating the change in absolute heart rate (b·min^−1^) 30 s (ΔHRR_30_ = HR_0_ − HR_30_), 60 s (ΔHRR_60_ = HR_0_ − HR_60_), 120 s (ΔHRR_120_ = HR_0_ − HR_120_), and 180 s (ΔHRR_180_ = HR_0_ − HR_180_) post-exercise during each week (W1, W6, W15), according to methods previously described in the literature [9]. Previous research has also demonstrated good reliability of ΔHRR values collected after submaximal exercise (ICC = 0.69) [34], with a typical error of 8 b·min^−1^ reported in the literature [35].

### 2.4. Statistical Analyses

A multivariable linear regression model, with random effects and nested repeated measures, was used to identify potential changes in the HRR response during entire recovery window (%MHR_0_, %MHR_15_, %MHR_30_, %MHR_45_, %MHR_60_, %MHR_120_, %MHR_180_) across the duration of the training academy (W1, W6, W15). Due to the previously identified influence of age [36] and BF [37] on HRR responses and known significant reductions in BF during the course of a firefighter recruit training academy [18], the multivariable linear regression model was adjusted for age and BF at each week as continuous covariates. Separate one-way analyses of covariance (ANCOVAs) and follow-up simple effects were also conducted to examine for potential differences in %MHR between weeks (W1, W6, and W15) at each interval of the recovery window (%MHR_0_, %MHR_15_, %MHR_30_, %MHR_45_, %MHR_60_, %MHR_120_, %MHR_180_). The potential influence of age and BF were controlled for by including these continuous variables as covariates in all one-way ANCOVAs.

In addition, to specifically examine potential changes in PSNS reactivation among firefighter recruits between weeks, one-way repeated measures ANCOVAs were utilized to identify potential differences in ΔHRR_30_, ΔHRR_60_, and ΔHRR_120_, and ΔHRR_180_ across time (W1, W6, W15). The potential influence of age and change in BF were controlled for by including these continuous variables as covariates in all repeated measures ANCOVAs.

Normality of data was ensured via visual inspection of distribution histograms associated with each variable. All descriptive data are reported as mean (SD) and all statistical analyses were conducted using SAS version 9.4 software (SAS Institute, Cary, NC, USA). An alpha of 0.05 was used to determine statistical significance for all analyses.

## 3. Results

**Figure 1:**
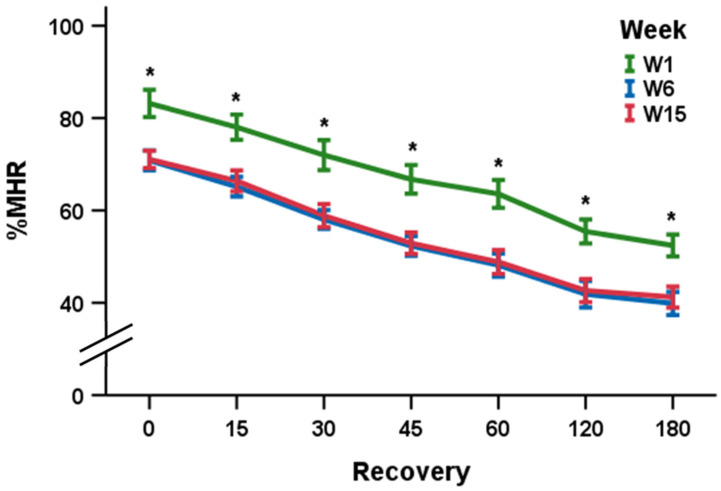
Changes in mean (± 95% confidence intervals) percent of age-predicted heart rate maximum (%MHR) during heart rate recovery (HRR) responses among firefighter recruits during firefighter training academy. * %MHR at week 1 (W1) is significantly (*p* < 0.001) higher than %MHR at week 6 (W6) and week 15 (W15).

After controlling for age (*F*_1,24.8_ = 0.46, *p* = 0.504) and BF (*F*_1,53.1_ = 17.77, *p* < 0.001), the multivariable linear regression model did not identify a statistically significant Week × HRR interaction (*F*_2,152_ = 0.31, *p* = 0.730), indicating the slope of the HRR responses during the entire recovery window did not significantly differ between W1, W6, and W15 (Figure 1).

However, one-way ANCOVAs identified significant differences in %MHR observed at %MHR_0_ (*F*_2,70_ = 34.59, *p* < 0.001), %MHR_15_ (*F*_2,70_ = 34.62, *p* < 0.001), %MHR_30_ (*F*_2,70_ = 37.16, *p* < 0.001), %MHR_45_ (*F*_2,70_ = 46.17, *p* < 0.001), %MHR_60_ (*F*_2,70_ = 45.99, *p* < 0.001), %MHR_120_ (*F*_2,70_ = 30.03, *p* < 0.001), and %MHR_180_ (*F*_2,70_ = 31.35, *p* < 0.001) (Table 2). Follow-up simple effects indicated that %MHR at W1 was significantly (*p*s < 0.001) higher than %MHR at both W6 and W15 at each interval of the recovery window (%MHR_0_, %MHR_15_, %MHR_30_, %MHR_45_, %MHR_60_, %MHR_120_, and %MHR_180_). No significant differences in %MHR were identified between W6 and W15 (*p*s > 0.05). These results indicate that the cardiovascular response associated with this sub-maximal task was significantly reduced at W6, but no further reductions were evident at W15.

Finally, results of the one-way repeated measures ANCOVAs did not identify significant differences in ΔHRR_30_ (*F*_2,44_ = 1.93, *p* = 0.157), ΔHRR_60_ (*F*_2,44_ = 1.34, *p* = 0.272), ΔHRR_120_ (*F*_2,44_ = 0.08, *p* = 0.921), or ΔHRR_180_ (*F*_2,44_ = 0.12, *p* = 0.888) across time (Table 3). These results indicate that no changes in the post-exercise PSNS reactivation of firefighter recruits were elicited during the training.

## 4. Discussion

The purpose of the current study was to examine longitudinal changes in HRR responses of firefighter recruits as they progress through their firefighter academy training program. Results of this study suggest that the physiological intensity of the step test task was reduced during the course of the recruit training program, as evidence by the reductions in %MHR between weeks, with significantly higher %MHR observed during W1 (vs. W6 and W15) at every interval of the recovery window. These reductions in physiological response demonstrated after submaximal exercise are consistent with the cardiorespiratory adaptations experienced as a result of chronic aerobic training (e.g., increased maximal aerobic capacity [V̇O_2_ max], increased arterial-venous oxygen [a-vO_2_] difference, increase stroke volume, etc.) [38]. Such adaptations would allow for a reduced cardiovascular load placed on the individual at the same given intensity, including during firefighting activities [39]. These positive adaptations in aerobic fitness are also consistent with previous literature demonstrating improvements in V̇O_2_ max among firefighter recruits throughout their training academies [18,19,20].

However, significant reductions in %MHR were only apparent from W1 to W6, with no further statistically significant reductions from W6 to W15, suggesting that positive cardiorespiratory physiological adaptations were not elicited throughout the entire recruit training program, which is also consistent with previous literature [18,19]. These findings are important as decreased aerobic fitness is associated with all-cause mortality and cardiovascular disease risk in the general population [40] and increased injury risk within the firefighter population specifically [21]. Therefore, future research should examine how different training programming may yield superior improvements in aerobic fitness of firefighter recruits throughout the entire training academy.

While differences in %MHR were identified between weeks, no differences in the slope of the HRR responses was observed between weeks. When combined with the lack of significant changes in ΔHRR_30_, ΔHRR_60_, ΔHRR_120_, and ΔHRR_180_ variables, these results indicate that the training academy did not significantly improve the PSNS reactivation of firefighter recruits. Given the “antiarrhythmic effect” produced by greater PSNS control [16] and the contribution of arrhythmias to firefighter SCDs [17], these collective findings suggest that even though positive physiological adaptations were elicited during the training academy, these adaptations may not create a cardio-protective effect to mitigate the increased risk of experiencing a SCD after returning from a fire call [8].

Although only a 3-min recovery window was examined, the results of the current study suggest that a training academy was not capable of improving the initial HRR response, or “fast phase”, which is largely attributed to PSNS reactivation [9]. However, it is possible that the training academy was capable of improving the “slow phase” of the HHR response (i.e., >3 min), which is considered to reflect SNS withdrawal [9]. Since recent research has also demonstrated a lack of physiological recovery even after 10 min of rest following submaximal exercise among active-duty firefighters [41], understanding if a training academy is capable of improving this element of the HRR response is still warranted. These results are important as current NFPA guidelines suggest that 10 min of recovery should be provided after exiting a fire [42].

Other research has utilized other methods of characterizing ANS recovery post-exercise among firefighters as well. In particular, by utilizing measures of heart rate variability (HRV), Ebersole et al. [41] identified a partial recovery of the ANS after submaximal exercise among active-duty firefighters within the first 3 min of recovery, which differed from HRR metrics collected. This disconnect is noteworthy as it has been theorized that HRR and HRV represent independent aspects of the PSNS activity via the vagus nerve in that HRR represents the reactivation of PSNS tone and HRV represents the modulation of the PSNS. As such, it is possible that the training academy was capable of improving other elements of post-exercise ANS recovery. Furthermore, although cardiorespiratory fitness was not assessed in the current study, previous research has recently identified a disconnect between cardiorespiratory fitness and post-exercise ANS recovery among firefighters utilizing measures of HRV as well. Specifically, Marcel-Millet et al. [43] did not identify a significant influence of V̇O_2_ max on the post-exercise HRV among firefighters. Therefore, further examination of the post-exercise ANS recovery of firefighter recruits throughout the completion of a training academy utilizing other measures of ANS function is warranted. Collectively, these results also highlight the need to better understand the impact of different physical training programs (e.g., aerobic training, high-intensity interval training, resistance training, etc.) on both the cardiorespiratory fitness and ANS recovery ability of firefighters.

### Strengths and Limitations

Although the current study had a small sample size (*n* = 25), there is still a paucity of longitudinal research within the firefighter scientific literature body, particularly describing changes among firefighter recruits during their training academy. Thus, the results of the current study provide unique and valuable data describing changes in HRR responses of a population underrepresented in the scientific literature (i.e., firefighter recruits). However, because all participants were members of the same firefighter academy training program, the results of this study are not generalizable to training academies associated with other non-urban fire departments (e.g., rural, wildland, etc.) or fire departments outside of the Midwest U.S. Similarly, due to the observational nature of the current study, the physical training programming within the training academy was not controlled by the researchers, and therefore, it was not possible to account for potential mediating factors influencing the observed HRR responses across time. In addition, the current study did not examine the HRR responses of firefighter recruits after maximal exercise. It is possible that adaptations created during the training academy only influenced the HRR responses of firefighter recruits after the completion of a highly strenuous activity. Finally, no females were included in the current study as there were no females enrolled in the training academy at that time. As such, future research should include larger sample sizes to examine for potential changes in HRR responses of both male and female firefighter recruits as they progress through their training programs and compare these changes across a variety of fire departments and submaximal and maximal exercise paradigms, while simultaneously controlling for and/or identifying factors that may mediate longitudinal changes in HRR responses.

## 5. Conclusions

The results of the current study indicate that although a 16-week training academy was capable of eliciting significant reductions in the cardiovascular demand placed among firefighter recruits after a bout of sub-maximal activity, likely due to positive adaptations in cardiorespiratory fitness, differences in post-exercise PSNS reactivation were not identified across time. Since it is hypothesized that greater PSNS control after exercise may create cardio-protective “antiarrhythmic effects”, these adaptations may not mitigate the increased risk of experiencing a SCD after returning from a fire. Therefore, further attention should be placed on understanding the changes in the ANS function of firefighter recruits, particularly as they transition to active-duty service, by both researchers and practitioners alike.

## Figures and Tables

**Table 1:** Participant characteristics at baseline, *n* = 25.

Variables	Mean (SD)	Range
Age, yrs	31.6 (4.4)	25–45
Height, cm	178.5 (5.2)	168.9–189.2
Body Mass, kg	89.3 (11.4)	70.8–114.8
BMI, kg·m^−2^	28.0 (3.4)	21.9–36.6
BF, %	14.8 (3.0)	10.9–21.6

BMI, body mass index; BF, percent body fat.

**Table 2:** Changes in HRR responses during the firefighter recruit training academy, *n* = 25.

Variable	W1	W6	W15
%MHR_0_	83.2 (7.2) *	70.8 (5.2)	71.1 (4.6)
%MHR_15_	78.0 (6.6) *	65.2 (5.2)	66.4 (5.5)
%MHR_30_	72.0 (7.8) *	58.1 (4.9)	58.9 (6.1)
%MHR_45_	66.7 (7.5) *	52.3 (5.6)	52.9 (5.6)
%MHR_60_	63.6 (7.3) *	48.2 (6.0)	48.8 (6.3)
%MHR_120_	55.5 (6.3) *	41.9 (7.0)	42.7 (6.1)
%MHR_180_	52.4 (5.7) *	39.8 (6.1)	41.2 (5.5)

HRR, heart rate recovery; W1, week 1; W6, week 6; W15, week 15; %MHR, percent of age-predicted maximal heart rate; %MHR_0_, percent of age-predicted maximal heart rate at 0 s; %MHR_15_, percent of age-predicted maximal heart rate at 15 s; %MHR_30_, percent of age-predicted maximal heart rate at 30 s; %MHR_45_, percent of age-predicted maximal heart rate at 45 s; %MHR_60_, percent of age-predicted maximal heart rate at 60 s; %MHR_120_, percent of age-predicted maximal heart rate at 120 s; %MHR_180_, percent of age-predicted maximal heart rate at 180 s. * W1 significantly greater than W6 and W15 (*p* < 0.001). Note: data presented as mean (SD).

**Table 3:** Changes in HRR metrics during the firefighter recruit training academy, *n* = 25.

Variable	W1	W6	W15
ΔHRR_30_, b·min^−1^	20.8 (6.3)	23.8 (9.4)	22.7 (6.2)
ΔHRR_60_, b·min^−1^	36.4 (6.9)	42.2 (9.8)	41.4 (8.7)
ΔHRR_120_, b·min^−1^	51.6 (8.7)	53.9 (11.8)	52.8 (8.8)
ΔHRR_180_, b·min^−1^	57.2 (10.1)	57.6 (10.8)	55.5 (8.5)

HRR, heart rate recovery; W1, week 1; W6, week 6; W15, week 15; ΔHRR_30_, 30-s heart rate recovery; ΔHRR_60_, 60-s heart rate recovery; ΔHRR_120_, 120-s heart rate recovery; ΔHRR_180_, 180-s heart rate recovery. Note: data presented as mean (SD).

## Data Availability

The data presented in this study are available on request from the corresponding author.
